# P31 - Natural history of wheat allergy in Greek children

**DOI:** 10.1186/2045-7022-4-S1-P86

**Published:** 2014-02-28

**Authors:** Stavroula Giavi, Paraskevi Korovessi, Nikolaos Douladiris, Emmanouil Manousakis, Nikolaos G Papadopoulos

**Affiliations:** 1 Allergy Clinic, 2nd Pediatric Clinic, University of Athens, Athens, Greece

## Background

Wheat allergy is one of the most common food allergies in children, yet few data are available regarding its natural history.

## Objectives

To define the natural course of wheat allergy in Greek children and identify factors that may predict outcome.

## Methods

We completed a retrospective medical record review of patients from the Allergy Clinic, 2nd Pediatric Clinic, University of Athens that were diagnosed as having wheat allergy. Patients were included in the study if they had a history of symptomatic reaction to wheat and or a positive wheat IgE test result.

Clinical history, laboratory results, and final outcome were recorded for 70 patients. Resolution of wheat allergy was based on food challenge results. Total IgE, wheat IgE (f4), specific gluten IgE (f79) and the ratio f4/ lgE, (at presentation), were compared between children with active or resolved wheat allergy, 4 years after their first reaction, performing the non-parametric *Mann-Whitney* test.

## Results

Rates of resolution were 20% by 5 years, 35% by 10 years(determined by *Kaplan-Meier* survival curves). Higher total IgE, f4, f79 or f4/IgE levels (at presentation) were not strongly associated with poorer outcomes at 4 years. f79 (*p* = 0.095) and f4 (p = 0.063) slightly correlated with active allergy.

Median age of first reaction to wheat was 8 months. History of AD was present at 96% of all children. Children with still active wheat allergy accounted for the 72% of the population and 75% of them had no history of other food allergy. In contrast, all children with resolved wheat allergy had multiple food allergies.

## Conclusion

The mean age of resolution of wheat allergy is approximately 11 years in this population (95% CI : 9,6-12,3). In a significant percentage of patients, wheat allergy persists into adolescence. Many children outgrew wheat allergy with even the highest levels of specific wheat IgE. Monosensitization to wheat appears to be a risk factor for persistence.

